# *Cryptosporidium* spp. and *Giardia* spp. in feces and water and the associated exposure factors on dairy farms

**DOI:** 10.1371/journal.pone.0175311

**Published:** 2017-04-12

**Authors:** Roberta dos Santos Toledo, Felippe Danyel Cardoso Martins, Fernanda Pinto Ferreira, Jonatas Campos de Almeida, Liza Ogawa, Hannah Lia Ettiene Peruch Lemos dos Santos, Maíra Moreira dos Santos, Filipe Aguera Pinheiro, Italmar Teodorico Navarro, João Luis Garcia, Roberta Lemos Freire

**Affiliations:** 1 Departamento de Medicina Veterinária Preventiva (DMVP), Universidade Estadual de Londrina (UEL), Londrina, Paraná, Brasil; 2 Centro de Ciências Agrárias, Universidade Estadual Norte do Paraná (UENP), Bandeirantes, Paraná, Brasil; Academic Medical Centre, NETHERLANDS

## Abstract

The aims of this study were to verify the prevalence of *Cryptosporidium* spp. and *Giardia* spp. in animal feces and drinking water on dairy farms and to identify a possible relation between the exposure factors and the presence of these parasites. Fecal samples from cattle and humans and water samples were collected on dairy farms in Paraná, Brazil. Analysis of (oo)cysts in the feces was performed by the modified Ziehl-Neelsen staining and centrifugal flotation in zinc sulfate. Test-positive samples were subjected to nested PCR amplification of *the 18SSU* ribosomal RNA gene for identification of *Cryptosporidium* and *Giardia* and of the *gp60* gene for subtyping of *Cryptosporidium*. Microbiological analysis of water was carried out by the multiple-tube method and by means of a chromogenic substrate, and parasitological analysis was performed on 31 samples by direct immunofluorescence and nested PCR of the genes mentioned above. Identification of the species of *Cryptosporidium* was performed by sequencing and PCR with analysis of restriction fragment length polymorphisms. The prevalence of *Giardia* and *Cryptosporidium* was higher in calves than in adults. Among the samples of cattle feces, *Cryptosporidium parvum* was identified in 41 (64%), *C*. *ryanae* in eight (12.5%), *C*. *bovis* in four (6.3%), *C*. *andersoni* in five (7.8%), and a mixed infection in 20 samples (31.3%). These parasites were not identified in the samples of human feces. Thermotolerant coliform bacteria were identified in 25 samples of water (45.5%). *Giardia duodenalis* and *C*. *parvum* were identified in three water samples. The *gp60* gene analysis of *C*. *parvum* isolates revealed the presence of two strains (IIaA20G1R1 and IIaA17G2R2) in the fecal samples and one (IIaA17G2R1) in the water samples. The presence of coliforms was associated with the water source, structure and degradation of springs, rain, and turbidity. The prevalence of protozoa was higher in calves up to six months of age. *C*. *parvum* and *G*. *duodenalis* were identified in the water of dairy farms, as were thermotolerant coliforms; these findings point to the need for guidance on handling of animals, preservation of water sources, and water treatment.

## Introduction

Water is an important vehicle for pathogens responsible for diarrhea worldwide [1]. Despite the recognition that the best quality of water (for human consumption) reduces the incidence of diseases, in Brazil, the water supply of small towns and rural areas differs from that in large urban centers [2]. In rural areas, ~70% of households consume water from alternative sources, with unmonitored potability [3].

Bacteria and protozoa are often primarily responsible for waterborne-illness outbreaks, but *Giardia* spp. and *Cryptosporidium* spp. have been the main cause of this problem in recent decades [4,5,6,7]. These protozoans are gastrointestinal parasites of vertebrates, including humans, and the life cycle is completed in a single host with the production of cysts and oocysts: environmental stages excreted with feces [8,9,10]. Thus, the fecal-oral transmission route facilitates the infection of humans by different vehicles contaminated by animal and human feces, especially water sources [11,12]. Developed countries have efficient systems for reporting and investigating of outbreaks of waterborne diseases, which is not the case in developing countries, where this information is obtained from research results reported in the scientific literature [4,7].

Cattle, especially calves, are an important source of infection for humans; the calves are reservoirs of *Cryptosporidium parvum*, a species with the zoonotic potential [8,9,13]. By analyzing the *gp60* gene of *C*. *parvum*, researchers have identified zoonotic strains in cattle feces and demonstrated the importance of these animals as sources of environmental contamination and of human infections [14,15,16]. Cattle are also reservoirs of *Giardia duodenalis*, and although they are most commonly infected with assemblage E of *G*. *duodenalis*, cattle have also been reported to be infected with zoonotic assemblage A and, occasionally, B [17,18,19,20]. Calves play an important role in environmental contamination, by excreting large quantities of (oo)cysts into the environment; these (oo)cysts can contaminate water sources used for human and animal consumption [4,21,22]. The contamination of water sources by these protozoa is of great importance in public health, since conventional methods of water treatment reduce these parasites, but do not completely eliminate them [23].

Due to the need for better understanding of the importance of cattle in the contamination of water sources on farms, the aim of this study was to determine the prevalence and to identify the species of *Cryptosporidium* and *Giardia* in fecal samples and in the water for human and animal consumption on dairy farms. Another aim was to find possible associations of exposure factors with the presence of these parasites.

## Materials and methods

### Study location and population

**Fig 1:**
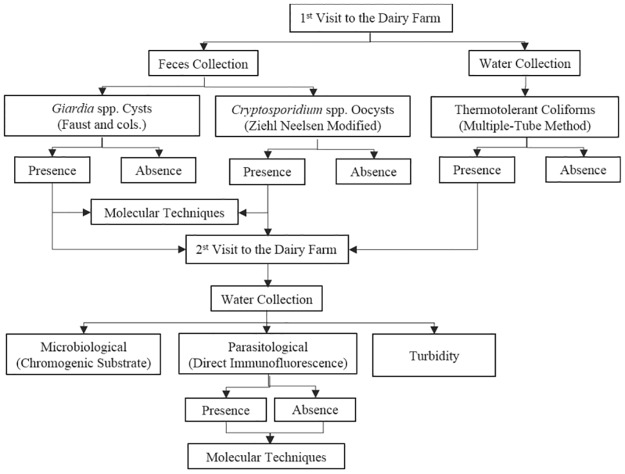
A flowchart of retrieval and analysis of fecal and water samples from 55 dairy farms in Paraná, Brazil, during 2012–2014.

This study was conducted in the cities of Campo Mourao and Araruna, western central Paraná, Brazil. The inclusion criteria were as follows: small family-own dairy farms assisted by the Paraná Institute of Technical Assistance and Rural Extension (EMATER) of Campo Mourao, totaling 55 farms: 20 in Araruna and 35 in Campo Mourao. The flowchart of the steps for retrieval and analysis of the stool and water samples in shown in Fig 1.

### Collection and initial analysis of the fecal samples

#### Collection of the samples

This study was approved by the Ethics Committee on Animal Experimentation (CEEA) of the Integrado College of Campo Mourao (approval # 242/2011) and by the Ethics Committee on Research Involving Humans (CEP-UEL) of the State University of Londrina (approval # 277/2011). The collections of water and feces from the animals were performed after written consent of the owners, through a science and authorization term approved by the CEEA. The collections of informations and human feces were performed after written consent of the participants, or their legal guardians (in the case of minors under 18 years of age) through a free and informed consent term approved by the CEP.

We collected fecal samples from all cattle from 0 to 24 months and, randomly, from a half of the herd of dairy cows. The human feces were collected from residents of the dairy farms who agreed to participate in the parasitological survey. The fecal samples of animals and humans were kept refrigerated until the analysis (performed within 24 h).

#### Analysis by optical microscopy

The fecal samples were diluted in distilled water, filtered and centrifuged, and the precipitate was used to find cysts and oocysts of protozoans. The presence of *Cryptosporidium* oocysts and *Giardia* cysts was verified by the modified Ziehl-Neelsen staining method [24] and centrifugal flotation in a 33% zinc sulfate solution [25], respectively. All test-positive fecal samples were stored in 2.5% potassium dichromate (final concentration) at 4°C for further molecular analysis.

### Collection and initial analysis of water samples

#### Collection of samples

Water samples were collected from human or animal sources in two visits. During the first visit, the collection of water was carried out at two points of each water source: one in the place of origin of the water and the other from the faucet. Approximately 250 mL of water was collected into a sterile bottle [26] in order to verify the presence of coliforms. During the second visit, new water collection was performed only on farms that showed contamination with fecal coliforms in water and/or the presence of (oo)cysts of *Cryptosporidium* and/or *Giardia* in fecal samples of cattle or humans obtained during the first visit (Fig 1). At this second stage, we performed new collection of water, for analysis of coliforms, and 10 L were collected into plastic containers for identification of *Giardia* and *Cryptosporidium*.

#### Microbiological analyses and turbidity

The water samples obtained during the first and second visits were subjected to microbiological analysis by the multiple-tube method and the chromogenic substrate method, respectively [27]. Samples from the second visit were also subjected to turbidity analysis on a HACH 2100Q turbidimeter (HACH^®^, Loveland, CO, EUA).

#### Concentration of water and the IFA

For concentration of the water samples, 3 L of each 10 L sample obtained during the second visit was filtered through cellulose ester membranes with 47 mm diameter and porosity 1.2 μm (Millipore^®^, Merck, Darmstadt, Germany), in a vacuum pump system [28]. In samples with high turbidity, where it was not possible to perform membrane filtration, the flocculation method of calcium carbonate was employed for water concentration [29]. We used 10 μL aliquots of each sample to perform a direct immunofluorescence assay *(IFA)* using the Merifluor *Cryptosporidium*/*Giardia* commercial kit (Merifluor^®^, Meridian Bioscience, Cincinnati, OH, USA). The criteria for identification and the enumeration of the (oo)cysts present in the water samples were described by our colleagues [30,31].

### Molecular analyses in feces and water samples

#### Nested PCR analysis for *Giardia* spp. and *Cryptosporidium* spp

All the samples were subjected to DNA extraction using the commercial kit (NucleoSpin^®^ Tissue, Macherey-Nagel, Düren, Germany).

To detect the presence of *Giardia* spp., fragments of the 18S ribosomal RNA (rRNA) were amplified using nPCR [32,33]. The samples were processed in triplicate; each sample contained 1× PCR Buffer, 200 μM each dNTP, 1.5 mM MgCl_2_, 400 nM each primer, 5% of dimethyl sulfoxide, 1.25 U of Platinum Taq DNA Polymerase, 1.5 μL of the extracted DNA, and ultrapure water. The amplification conditions were as follows: 5 min at 95°C; followed by 35 cycles of 45 s at 94°C, 45 s at 58°C in first reaction and 45 s at 55°C in the second reaction, and 60 s at 72°C; with 5 min at 72°C as the final extension step.

To detect *Cryptosporidium* spp., fragments of the 18S rRNA gene were amplified using nPCR [34]. The samples were processed in triplicate, and each reaction mixture contained 1× PCR Buffer, 200 μM dNTP, 2.5 mM MgCl_2_, 400 nM each primer, 1.25 U of Platinum Taq DNA Polymerase, 2.0 μL of the DNA extracted from each sample, and ultrapure water. The material obtained in the first reaction was diluted with 50 mL before use in the second reaction. The amplification conditions for both the first and second reaction were as follows: 5 min at 95°C; followed by 35 cycles of 45 s at 94°C, 45 s at 55°C, and 60 s at 72°C; with the final extension step 5 min at 72°C.

The PCR products were subjected to electrophoresis in a 1.5% agarose gel (UltraPure^™^ Agarose, Invitrogen, Waltham, MA, USA) stained with DNA gel stain (SYBR^®^ Safe, Invitrogen, Waltham, MA, USA) and visualized with ultraviolet light.

#### DNA sequencing

This procedure was performed on all water samples positive for *Giardia* spp. and all fecal and water samples positive for *Cryptosporidium*. Sequencing was performed using an ABI3500 sequencer genetic analyzer (Applied Biosystems, Life Technologies^™^, Carlsbad, CA, USA). The resulting nucleotide sequences were compared with the standard *Cryptosporidium* and *Giardia* sequences in GenBank by means of the Basic Local Alignment and Search Tool (BLAST) and by manual alignment in the BioEdit software (Biological Sequence Alignment Editor).

#### PCR with restriction fragment length polymorphism analysis of *Cryptosporidium*

Samples positive for *Cryptosporidium* (in the nPCR analysis) that yielded more than one gene fragment in the amplified sequence were subjected to genetic characterization by means of Restriction Fragment Length Polymorphism Analysis (RFLP). To identify the species in a sample, the products obtained in the second reaction of the nPCR were cleaved with restriction enzymes *Ssp*I, *Ase*I, *Mbo*II, and *Dde*I [35,36]. The reaction was performed with 5 μL of DNA, 2 μL of a restriction buffer, 3 IU of the enzyme (New England Biolabs, Ipswich, MA, USA), and ultrapure water. The digestion was performed at 37°C for 1 h, and the products were subjected to electrophoresis in a 2.5% agarose gel stained with SYBR^®^ Safe.

#### The gp60 gene of *C*. *parvum*

For subtyping of *C*. *parvum* isolates, we performed nPCR on one fragment (832 bp) of the *gp60* gene [37]. The first reaction was composed of 1× PCR Buffer, 200 μM dNTP, 2.5 mM MgCl_2_, 400 nM each primer, 1.25 U of Platinum Taq DNA Polymerase, 2.0 μL of the extracted DNA, and ultrapure water. In the second reaction, the same concentrations were used, but 1.0 μL of the material obtained in the first reaction was used instead of the DNA sample. The amplification conditions for both the first and the second reaction were the following: 3 min at 95°C; followed by 35 cycles of 45 s at 94°C, 45 s at 50°C, and 1 min at 72°C; with final extension for 10 min at 72°. The amplicons were subjected to electrophoresis, purified, and sequenced as described above.

### Rainfall

To compare the results from the samples to seasonal variables, the water precipitation data 24 and 48 h before collection were obtained from the Paraná Meteorological System (SIMEPAR).

### Epidemiological and statistical analysis

The farmers were interviewed using an epidemiological questionnaire containing variables for the type of production, rural sanitation, and health and individual characteristics of the animals and humans; these variables were analyzed for association with the presence/absence of fecal coliforms in the water and *Cryptosporidium* spp. and *Giardia* spp. in feces and water. The comparison between the frequency data from the analysis of feces and water and the variables of the epidemiological survey was performed using the chi-squared test or Fisher’s exact test. The magnitude of the association was determined by calculating the odds ratio (OR). The calculations were performed in the EpiInfo^™^ software, version 3.5.2 (CDC, Atlanta, GA, USA), with the level of statistical significance at 5%.

## Results

### Analyses of cattle fecal samples

We collected fecal samples from 937 heads of cattle: 558 in Campo Mourao and 379 in Araruna. According to optical microscopy (OM), 71 (7.6%) stool samples were positive for *Giardia* spp. cysts from 27 (49.1%) farms and, among the positive samples according to OM, 48 also positive in the nPCR for the 18S rRNA gene. *Cryptosporidium* spp. were identified in 96 (10.2%) samples from 37 (67.3%) farms, when analyzed by OM, and among these, 64 samples were also positive for the 18S rRNA gene (Table 1). The prevalence was higher among calves up to 6 months of age, and among them, was higher in the age group 0–2 months old for both *Giardia* spp. and *Cryptosporidium* spp. (Table 1).

**Table 1:** Prevalence of *Giardia* spp and *Cryptosporidium* spp in feces of cattle from 55 dairy farms in Paraná, Brazil, from 2012 to 2014.

Age Group (months)	Number of samples	*Giardia* spp.		*Cryptosporidium* spp.	
		OM (%)	*n*PCR 18S (%)^a^	OM (%)	*n*PCR 18S (%)^a^
0–2	146	25 (17.1)	15 (60)	37 (25.3)	34 (91.9)
2–4	133	15 (11.3)	13 (86.7)	9 (6.8)	7 (77.8)
4–6	99	6 (6.1)	5 (83.3)	8 (8.1)	6 (75)
Total (0 a 6)	378	46 (12.2)	33 (71.7)	54 (14.3)	47 (87)
6–12	81	6 (7.4)	6 (100)	5 (6.2)	3 (60)
12–24	54	0	0	6 (11.1)	1 (16.7)
>24	424	19 (4.5)	9 (47.4)	30 (7.1)	13 (43.3)
Total (> 6 months)	559	25 (4.8)	15 (2.7)	41 (7.3)	17 (3.0)
Total (All)	937	71 (7.6)	48 (67.6)	96 (10.2)	64 (66.7)

^**a**^ Percentage calculated using the number of positive data from optical microscopy (OM). nPCR: nested PCR, 18S: 18S rRNA gene.

Sequence analysis of the 18S rRNA gene was successful in 34 of the 64 samples positive for *Cryptosporidium* according to nPCR. Among them, 27 (42.2%) showed 100% similarity to *C*. *parvum*, five (7,7%) to *C*. *ryanae* and two (3,1%) to *C*. *bovis* (GenBank: KX929930 to KX929963).

In 20 (31.1%) sequenced samples, there were overlapping gene fragments in the electropherogram. The species that were identified by genetic sequences in various age groups of the cattle are shown in Table 2.

**Table 2:** Distribution of *Cryptosporidium* species by age groups according to genetic sequencing of fecal samples from 64 heads of cattle and PCR-RFLP of fecal samples from 15 heads of cattle from 28 dairy farms in Paraná, Brazil, from 2012 to 2014.

Age Group (months)	DNA sequencing (%)						PCR-RFLP^b^ *Cryptosporidium* spp. (%)				
	Positive Samples (*n*PCR^a^)	*C*. *parvum*	*C*. *ryanae*	*C*. *bovis*	ND ^c^	Fragments of overlap	*C*. *parvum/C*. *bovis*	*C*. *parvum/C*. *ryanae*	*C*. *parvum/C*. *andersoni*	Single species	ND
0–2	33	16	0	0	4	13	2	2	0	5^d^	4
2–4	8	4	1	0	1	2	0	1	1	0	0
4–6	8	4	1	1	2	0	0	0	0	0	0
6–12	3	1	0	0	0	2	0	0	1	1^e^	0
12–24	0	0	0	0	0	0	0	0	0	0	0
>24	12	2	3	1	3	3	0	0	3	0	0
Total	64	27 (42.2)	5 (7.7)	2 (3.1)	10 (15.6)	20 (31.3)	2 (3.1)	3 (4.7)	5 (7.8)	6 (9.4)	4 (6,3)

^**a**^nPCR: nested PCR,

^**b**^PCR-RFLP: restriction fragment length polymorphism,

^**c**^ ND: species not determined (ND) due to illegible sequence (sequencing data) or absence of DNA (PCR-RFLP),

^**d**^*C*. *parvum*,

^**e**^*C*. *bovis*.

The PCR-RFLP analysis of 20 samples with overlapping fragments allowed us to identify two samples positive for *C*. *parvum* and *C*. *bovis*, three for *C*. *parvum* and *C*. *ryanae*, five for *C*. *parvum* and *C*. *andersoni*, five only for *C*. *parvum*, and one only for *C*. *bovis*. Four cattle samples could not be analyzed by PCR-RFLP due to the absence of DNA (Table 2). The samples of cattle with a likely mixed infection belonged to 16 farms, 15 of which kept animals of different ages in the same environment.

Among the 42 samples that tested positive for *C*. *parvum*, 20 underwent nPCR amplification of *gp60* gene, all calves. The sequencing analysis in 16 of these samples was successful. Among the samples analyzed, we identified two subtypes that showed 100% of identity with subtypes IIaA17G2R1 and IIaA20G1R1 (GenBank: KY073279 to KY073281).

Among the variables analyzed, the production system, the number of cows greater than 40, feeding of cows from the trough, frequent diarrhea on the farm, diarrheal stool samples, and age up to 6 months were significantly associated with the presence of *Giardia* cysts in the fecal samples. The European breed, diarrheal stool samples, and age up to 6 months were associated with the presence of *Cryptosporidium* oocysts in feces (Table 3).

**Table 3:** Variables with a statistically significant association with the presence of cysts of *Giardia* spp. and/or oocysts of *Cryptosporidium* spp. in fecal samples from 937 heads of dairy cattle in Paraná, Brazil, from 2012 to 2014.

**Exposure variables**	**Cysts of *Giardia* spp.**	**OR**^b^**(95% CI**^c^**)**	***p*** ^d^
	**Positive samples/Total (%)**		
**Production system**			
Business	10/63 (15.9)	2.51^a^(1.08–5.31)	0.0318
Family	61/874 (7.0)		
**Lactating Cows**			
1 to 40	53/801 (6.6)	0.46 (0.26–0.82)	0.0117
>40	18/136 (13.2)		
**Feeding of Cows**			
Only trough	10/44 (22.7)	4.02^a^(1.68–8.78)	0.002
Trough and pasture	61/893 (6.8)		
**Diarrhea frequency**			
High	41/346 (11.8)	2.51(1.54–4.11)	0.0003
Low	30/591 (5.1)		
**Characteristics of feces (calves up to 2 months old)**			
Liquid/pasty	11/35 (31.4)	3.18 (1.28–7.87)	0.0203
Firm	14/111 (12.6)		
**Age Group (All)**			
0–6 months	46/377 (12.2)	2.97 (1.79–4.93)	0.0001
>6 months	25/560 (4.5)		
**Age Group (up to 6 months)**			
0–2 months	25/146 (17.1)	2.08 (1.12–3.87)	0.0296
2–6 months	21/232 (9.1)		
**Exposure variables**	**Oocysts of *Cryptosporidium* spp.**	**OR (95% CI**^c^**)**	***p*** ^d^
	**Positive samples/Total (%)**		
**Breed**			
European	78/646 (12.1)	2.21 (1.29–3.81)	0.005
Zebu or Cross-bred Zebu	17/291 (5.8)		
**Characteristics of feces**			
Liquid/pasty	23/98 (23.5)	3.31 (1.96–5.61)	0.0001
Firm	72/839 (8.6)		
**Age Group (All)**			
0–6 months	54/95 (56.8)	2.12 (1.38–3.25)	0.0007
>6 months	41/559 (7.3)		
**Age Group (up to 6 months)**			
0–2 months	37/146 (25.3)	4.29 (2.31–7.97)	0.0001
2–6 months	17/232 (7.3)		

^**a**^Fisher's exact test;

^**b**^OR: odds ratio;

^**c**^CI: confidence interval;

^**d**^*p*: probability.

### Analyses of the human fecal samples

We collected fecal samples from 83 humans, among them, 15 (18.3%) were positive for at least one parasite. *Endolimax nana* was identified in six (40%) samples, *Entamoeba coli* in four (26.7%), *Iodamoeba bütschlii* in three (20%), and *Entamoeba histolytica* in two (13.3%). The age group with the highest prevalence of parasitism was 0–12 years: six positive samples (35.3%). No samples were positive for *Cryptosporidium* spp. and *Giardia* spp.

### Analyses of the water samples

Among the 55 farms visited, in 48 (87.3%), the water sources for human and animal consumption were the same, and 27 (49.1%) were springs.

During the first visit to the farms, we collected 124 samples from 62 sources. Among these, 50 samples (40.3%) and 27 sources (43.5%) were positive for thermotolerant coliforms on 25 (45.5%) farms. During the second visit, a water sample was collected at sources totaling 31 samples; in 15 of them (48.4%), thermotolerant coliforms were detected (Table 4).

**Table 4:** Prevalence of thermotolerant coliforms in 155 water samples obtained during the first visit and second visit to dairy farms in Paraná, Brazil, from 2012 to 2014.

Water sources	First Visit			Second Visit		
	Number of sources	Number of samples	Positive Samples, TTC^a^ (%)	Number of sources	Number of samples	Positive Samples, TTC^b^ (%)
Spring	31	62	35 (56.5)	24	24	12 (50)
Artesian well	20	40	3 (7.5)	2	2	0
Shallow well	8	16	6 (37.5)	2	2	0
River	3	6	6 (100)	3	3	3 (100)
Total	62	124	50 (40.3)	31	31	15 (48.4)

^**a**^Thermotolerant coliforms according to the multiple-tube method;

^**b**^according to the chromogenic substrate method.

The four test-positive samples showed high turbidity and presence of thermotolerant coliforms and were collected after rainy days (Table 5). All samples positive for *Giardia* spp. and/or *Cryptosporidium* spp. in the IFA were also test-positive in the nPCR analysis. Among the test negative samples in the IFA, only one farm (number 27) yielded amplified DNA of *Cryptosporidium* during nPCR (Table 5).

**Table 5:** Springhead, turbidity, bacteriological parameters of water samples positive for *Cryptosporidium* or *Giardia* species identified by genetic sequencing, and rainfall data 24 and 48 h prior to collection on four dairy farms in Paraná, Brazil, in 2014.

Property	Source of water	*Giardia* spp.				*Cryptosporidium* spp.				Turbidity (NTU)^h^	TTC^i^ (MPN^j^/100mL)	Rainfall prior to collection (mm)	
		IFA^b^ (Cy*/L)*^d^	*n*PCR^f^	Sequencing	Positive Cattle	IFA^b^ (Oo*/L*^c^*)*	*n*PCR^f^	Sequencing/PCR-RFLP^g^	Positive Cattle			24 h	48 h
26	Spring	0	(-)	-	Yes	1.3	(+)	*C*. *parvum*	Sim	36.4	1046	83	76
27 ^a^	River	12	(+)	ND^e^	No	0	(+)	*C*. *parvum/C*. *andersoni*	Não	98.5	2419.5	44	0
34	Spring	0.34	(+)	*G*. *duodenalis*	Yes	0.34	(+)	*C*. *parvum/C*. *andersoni*	Sim	25.6	24.5	43	0
44	Spring	2.1	(+)	*G*.*duodenalis*	Yes	0	(-)	-	Sim	64.47	2419.6	182.4	0

^**a**^Strongly concentrated by the flocculation method of calcium carbonate [29];

^**b**^IFA: direct immunofluorescence assay;

^**c**^Oo/L: oocysts/liter of water;

^**d**^Cy/L: cysts/liter of water;

^**e**^ND: Not determined due to illegible sequence;

^**f**^nPCR: nested PCR;

^**g**^PCR-RFLP: restriction fragment length polymorphism;

^**h**^NTU: nephelometric turbidity unit;

^**i**^TTC: thermotolerant coliforms;

^**j**^MPN: most probable number.

Sequencing analysis of the 16S rRNA gene of the three water samples positive for *Giardia* was successful in two, which showed 100% identity with *G*. *duodenalis* (accession # KJ867494.1). The sequencing analysis of the three water samples positive for *Cryptosporidium* spp. according to nPCR was successful in one sample, which showed 100% identity with *C*. *parvum* (GenBank: KX929941). The other two samples showed an overlap of the sequencing fragments and were subjected to PCR-RFLP, which revealed the presence of *C*. *parvum* and *C*. *andersoni* in both samples (Table 5).

Sequencing analysis of the *gp60* gene in three water samples positive for *C*. *parvum* was successful in two, where the identified subtype was IIaA17G2R1 (GenBank: KY073279 e KY073280). One of these samples belongs to farm 26, where we identified the same subtype in two calves younger than 30 d.

Among the variables analyzed, open sources like rivers and springs, collection of samples at the source, the absence of a cover and side protection at the source, rain in the last 48 h, and the absence of riparian vegetation near the springs were significantly associated with the presence of thermotolerant coliforms in water (Table 6).

**Table 6:** Variables with a statistically significant association with the presence of thermotolerant coliforms in 124 water samples from 55 dairy farms in Paraná, Brazil, from 2012 to 2014.

Variables	Thermotolerant Coliforms	OR^a^ (95% CI^b^)	*p*^c^
	Positive samples/Total (%)		
**Source of Water**			
River	6/6 (100)	-	0.0001
Spring	35/62 (56.45)	15.99 (4.45–57.45)	0.0001
Shallow well	9/60 (15)	7.4 (1.57–34.93)	0.01294
Artesian well	3/40 (7.5)	1	
**Collection site**			
Source	22/37 (59.46)	3.09 (1.4–6.85)	0.0084
Water tank/Tap	28/87 (32.2)		
**Slapping source**			
Yes	36/103 (34.95)	0.27 (0.08–0.8)	0.0074
No	14/21 (66.7)		
**Rainfall prior to collection (up to 48 h)**			
Yes	25/46 (54.3)	2.52 (1.2–5.34)	0.0241
No	25/78 (32.1)		
**Spring with side protection**			
Yes	18/40 (45)	0.24 (0.074–0.78)	0.02892
No	17/22 (77.3)		
**Spring with riparian forest**			
Yes	19/42 (45.2)	0.21 (0.06–0.72)	0.02107
No	16/20 (80)		

^**a**^OR: odds ratio;

^**b**^CI: confidence interval;

^**c**^*p*: probability.

Among the water samples obtained during the second visit to farms, we identified a significant association between turbidity above 5 UT and the presence of thermotolerant coliforms (p = 0.049; OR = 0.17; 95% confidence interval [CI] 0.035–0.79). The association of the samples positive for *Giardia* spp. and/or *Cryptosporidium* spp. with exposure variables evaluated in this study was not statistically significant, but the presence of these parasites was found in water samples with thermotolerant coliforms at >1000 most probable number (MPN) per 100 mL, turbidity above 5 UT, rainfall above 43 mm 24 h before the sample collection, the absence of riparian vegetation and of external protection, and closeness to pasture.

## Discussion

*Cryptosporidium* spp. are economically important enteropathogens that cause diarrhea in calves and can compromise development of the animal or kill it [38]. The prevalence of infections with *Cryptosporidium* spp. among cattle varies widely due, among other factors, to age differences among the animals. In studies that showed high prevalence, most of the sampled animals were younger than 6 months, and in the studies that showed lower prevalence, more than 50% of the sample consisted of animals older than six months, as is the case in our study [39,40,41,42,43]. When analyzed separately, the prevalence of *Cryptosporidium* spp. was significantly higher among calves up to 2 months old (25.3%; p = 0.0001) and up to 6 months old (14.3%; p = 0.0007) when compared with the calves older than 2 or 6 months, respectively. In a longitudinal study conducted in the USA [44], prevalence of cryptosporidiosis among 30 heads of cattle from birth to 2 years of age was inversely proportional to the age of the animals, findings consistent with the results of our study.

The prevalence of *Giardia* cysts in the feces of cattle was higher among younger calves in agreement with previous study [45]. Nonetheless, higher prevalence of *G*. *duodenalis* is commonly observed among postweaned calves when compared to preweaned ones or animals older than 12 months, owing to sudden changes in the management of these animals [18,45,46,47,48,49,50]. In our study, factors such as the type of establishment, the number of animals sampled, management conditions, and the environment may have interfered with the analysis, so that the highest prevalence was observed in the age group of preweaned calves.

The variation in the prevalence of bovine cryptosporidiosis and giardiasis reported in different studies within the same age group can also be caused by factors related to the management and characteristics of the animals. In our study, we demonstrated a significant association between the feeding of cows exclusively from the trough and the number of lactating animals greater than 40 with the presence of *Giardia* spp. in feces. Research groups have reported higher prevalence on farms with breeding of cows and calves in a closed collective environment and with more animals [43,51]. Closed collective environments are favorable for the maintenance of these parasites because of shading and humidity, keeping the infectivity for longer periods; furthermore, the higher the animal density in an environment, the greater the contamination, and consequently, the greater the ingestion of (oo)cysts [52]. In our study, there was greater prevalence of cryptosporidiosis in European-bred animals when compared to Zebu animals or animals cross-bred with Zebu. Other studies have shown that prevalence of cryptosporidiosis is similar in Zebu and European breeds, indicating that management practices in the livestock influence the parasitism more than a difference in breeds [41,53]. In our study, although most farms were characterized as a family business, those who possessed higher levels of technologies with higher stocking density, were breeding Holstein cows, whereas farms with lower stocking density worked with animals cross-bred with the Zebu breed. These characteristics may have influenced the association between cryptosporidiosis and the European breed, as demonstrated by other researchers [39].

Among the animals that had diarrhea, 23.5% also excreted oocysts of *Cryptosporidium* spp., with a significant association, but this rate increased when we considered only calves of 0 to 2 months of age (71.4%). Other studies have shown the high prevalence of this parasite in the feces of calves with diarrhea [50,54,55]. The immune system is immature in neonates; thus, changes in the intestinal mucosa are more pronounced than in older calves; this situation increases the morbidity of cryptosporidiosis [50]. In addition, the environment where the newborns remain promotes greater contact with feces, and consequently, higher intake of oocysts because cows are asymptomatic carriers of the parasite and excrete larger amounts of oocysts with feces in the farrowing period, thereby increasing environmental pollution and the risk of infection among neonates [52,56]. In extensive farming systems, the calves are moved to the pasture with age; thus, the above contact is reduced [38]. When we analyzed the association between diarrhea and excretion of *Giardia* spp. in all the animals, there was no statistically significant relation, and only 11 (12.6%) of the 97 animals with diarrhea also excreted cysts. Nevertheless, this frequency increased to 31.4% if we considered only the population of calves of 0 to 2 months of age, with a statistically significant association. Despite the uncertainty regarding the role of *Giardia* as a primary pathogen in cattle diarrhea, some studies have shown this possibility, in which were identified identified cysts of *Giardia* spp. in 36% of 669 fecal samples [57] and in 36.7% of 690 fecal samples [50] from cattle, and a significant association was observed between the presence of the parasite and diarrhea; this result is consistent with our findings. As is the case with other parasites (such as *Cryptosporidium* spp.), *Giardia* diarrhea is determined by several factors such as virulence of the parasite, the host immune response, and the infectious dose, the latter being strongly dependent on the management system, with the greatest influence in intensive farming systems [22].

In our study, there were differences in the amount of positive samples in optical microscopy and nested PCR. This may have occurred due to the method used to preserve the parasites, in potassium dichromate. The potassium dichromate are PCR inhibitor and extensive washes are required to remove it, wich may lead to a loss of cysts/oocysts thus reducing PCR sensitivity, especially in samples with small amount. Some samples collected had a small number of cysts / oocysts in the microscopic analysis, even after the concentration of these samples [58].

Judging by the results of sequencing and PCR-RFLP, *C*. *parvum* was the most frequent species in our study (64%), most notably among calves up to 6 months of age. In several studies around the world, *C*. *parvum* has been identified with higher prevalence among calves up to 2 months of age, with a considerable decrease in frequency above this age [44,59,60,61,62,63]. In the present study, the high prevalence of this species among calves older than 2 months may be a consequence of the handling of these animals on the farm because a large number of producers did not separate calves of different ages into different environments. A few samples were identified as *C*. *ryanae* and *C*. *bovis*, in line with other reports [36,61,64,65,66], but many researchers have detected these species more often when compared to *C*. *parvum* among cattle older than 2 months [44,53,59,66]. In others studies was suggested that infection with *C*. *bovis* or *C*. *ryanae* in animals (parasitized with greater severity by *C*. *parvum)* may be latent because PCR analysis of fragments of 18SSU rRNA identifies the predominant species [36,67,68]. *C*. *andersoni* was also identified in a few samples, mostly in animals older than 24 months. This species is mainly identified among heifers and adult animals [44,63,69], but some investigators in Brazil identified *C*. *andersoni* at a high frequency in young calves too [66,70]. In our study, the prevalence of *C*. *parvum* among the adult cattle was also higher than that of the other species; such data are scarce in the literature [71,72]. The high prevalence of this species among adult animals can contribute to infection of newborn calves, and consequently, increase environmental contamination [38].

The gp60 gene has a high degree of sequence polymorphism in C. hominis, C. meleagridis and C. parvum isolates, making it possible to identify these species in subtypes groups [68]. Groups IIa and IId of C. parvum have been identified in humans and ruminants, but the IIa family is the most commonly identified in cattle and humans worldwide [13]. Analysis of the *gp60* gene of *C*. *parvum* is a useful tool for identification of subtypes and zoonotic strains not widely reported in Brazil. In our study, we identified two strains of the IIa family in 16 stool samples: strains IIaA20G1R1 and IIaA17G2R1, which not been reported in studies in this same country. The most common strain in our study, IIaA20G1R1, has been detected in cattle in some regions of the world: Serbia and Montenegro, Sweden, and Argentina [73,74,75,76], but there are no reports of this strain in humans. The second most common strain identified in stool samples, IIaA17G2R1, has been detected in other studies on cattle [77,78] and on humans [79,80]. After an outbreak of human cryptosporidiosis at a camp in North Carolina, USA, the Centers for Disease Control and Prevention (CDC) have demonstrated that ~60% of the confirmed cases were caused by *C*. *parvum* strain IIaA17G2R1, and the same strain was present in the animal feces at the camp, suggesting that zoonotic transmission had taken place [80]. Calves infected with *Cryptosporidium* and *Giardia* spp. may excrete ~10^6^–10^7^ (oo)cysts per gram of feces, with higher excretion peaks among young animals, becoming intermittent with the development of adaptive immunity [38,81]. Therefore, the presence of calves infected with a zoonotic strain of *C*. *parvum* and/or *G*. *duodenalis* favors greater contamination of the immediate environment, and consequently, of the water; this state of affairs can increase the risk of human cryptosporidiosis and giardiasis [38].

On the visited farms, the main sources of drinking water for humans were underground sources, where the presence of fecal coliforms is relevant, especially in springs and shallow wells. The absence of riparian vegetation and of spring protective structures, such as raised edges and a cover, was significantly associated with the presence of thermotolerant coliforms in the water. A study conducted in the state of Goias, Brazil, revealed poor quality of water owing to high environmental degradation caused by a shortage of vegetation near the springs, animal trampling, and proximity to the areas of pasture and crops [82]. The vegetation around springs serves as a paperlike physical barrier between the ground and water, thereby reducing runoff and erosion, and thus the contamination of water [83]. Therefore, it is not surprising that higher turbidity (p = 0.0490) and precipitation preceding the collection of water samples (p = 0.0366) showed a significant association with the presence of thermotolerant coliforms in our study. This presence in six of the 16 samples collected from shallow wells did not show an association with the type of well structure because all had adequate protective caps, an outer wall above the ground, internal coating, and paving around the well. These characteristics have been identified as protective factors against contamination of shallow wells with thermotolerant coliforms in a study conducted in Mato Grosso do Sul, Brazil [84]. The risk of contamination of groundwater via the passage of microorganisms through the ground exists; this is because although layers of soil and rock decrease this risk, they are not capable of preventing contamination, particularly in agricultural areas with substantial soil degradation and dumping of animal feces [85].

The presence of *Cryptosporidium* spp. and/or *Giardia* spp. was demonstrated in four of the 31 water samples analyzed here, three of which came from springs and a river. The presence of protozoa in springs underscores the importance of protecting these water sources because these three sources had no riparian vegetation or adequate protective structures and were situated on the lower ground and close to grazing areas. Contamination of springs by these protozoa and its relation to protective structures and the water source environment have been demonstrated in other studies. In Campos do Jordão, SP, Brazil, was reported the presence of at least one of these protozoa in two springs in an urban area and in one spring in a rural area and identified a relation with poor plumbing, inadequate tanks, soil porosity, the presence of sewage, localization in lower parts of the land, and proximity to pastures [86]. In indigenous lands, Paraná, Brazil was reported the presence of these protozoa in 42.8%, 14.3%, and 14.3% of water samples collected from a river, springs, and artesian wells, respectively, and attributed their presence to contamination by human and animal feces [87]. The number of cysts (0.34–12/L) and oocysts (0.34–1.3/L) identified in the water samples is highly relevant to public health because a small dose of (oo)cysts is sufficient to cause a clinical infection [4]. Furthermore, the three contaminated sources identified in the present study were used for human consumption. Water samples positive for protozoa were collected after precipitation, and the concentration of (oo)cysts in the samples increased with turbidity. Although there was no significant association between these parameters and the presence of protozoa, some studies have established a positive correlation. In Australia was reported a positive and significant correlation between turbidity and the flow of water in samples positive for *Cryptosporidium* spp. collected in a river of multiple use [88]. In Viçosa, Minas Gerais, Brazil, was identified a high concentration of oocysts in drinking-water sources that were characterized by unprotected basins with intensive human settlement and agricultural activities; those authors inferred the influence of rainfall on the presence of these protozoa (not statistically significant) [89]. This correlation between turbidity and the presence of parasites also demonstrates the importance of the use of adequate techniques for the filtration of samples with high turbidity rates to monitor the contamination of water sources. In our study, in one of the samples, it was not possible to perform the concentration by filtration in membranes, and it was necessary to use another technique, in this case the flocculation method, which proved efficient but laborious. Other studies have demonstrated efficient methodologies to recuperate (oo)cysts in water samples with high turbidity, as the combination of polyester microbibre filters (ARAD) and a loop-mediated isothermal amplification (LAMP) to detect few (oo)cyst in large quantity of water with high turbidity [90]

Thermotolerant coliforms are key indicators of fecal contamination of water, and their presence is suggestive of the presence of other fecal microorganisms. In this study, the water sources positive for *Cryptosporidium* and/or *Giardia* spp. also showed high bacterial counts: three of them above 1000 MPN per 100 mL. Ordinance 2914/11 [91] establishes the necessity of monitoring of these protozoa at points of water capture when the annual geometric mean of *Escherichia coli* identified in the surface raw water is ≥1000 cells per 100 mL. Even though the high bacterial count identified in the samples of the present study represents a one-off result, it is important to recognize that strong fecal contamination of these sources poses a risk to human and animal health on these farms. In addition, ~70% of Brazilian rural municipalities use alternative sources of water supply, without treatment and monitoring of potability established by a relevant ordinance [3].

The relation between the presence of *Giardia* spp. in the samples and the presence of these protozoa in animal feces on these farms was impossible because this association was not detected by DNA sequencing of cattle fecal samples owing to low sensitivity in terms of identification of assemblages of the 18SSU gene used in this study [92]. *G*. *duodenalis* was identified in water samples this study, but markers for identification of assemblages were not used; therefore, it was not possible to establish the source of contamination [93,94]. All water samples where we detected cysts of *Giardia* spp. were from farms with animals infected by this parasite. Although assemblage E (not zoonotic) is most commonly identified in cattle, some studies have shown the prevalence of zoonotic-infection assemblages of 3–43% among animals in various categories; these data point to the importance of these animals for environmental contamination by zoonotic assemblages of parasites [18,20,46,46–48,95].

Our analysis of gene sequencing and PCR-RFLP analysis of *Cryptosporidium* isolates from water samples revealed the presence of *C*. *parvum* on three farms and *C*. *andersoni* on two. The species most commonly associated with diseases in humans are *C*. *hominis* (exclusively anthroponotic) and *C*. *parvum* (a zoonotic species most often found in rural areas) [16,96,97]. Other studies have shown an association of a high frequency of infection with *C*. *parvum* among humans with the presence of infected cattle near water catchment areas [72,98]. *C*. *parvum* has different hosts; therefore, its presence in water may be the result of contamination by feces of domestic and/or wild animals or by human sewage [43,99]. On the other hand, the presence of *C*. *andersoni* and *C*. *parvum* strain IIaA17G2R1 in water and feces of cattle on these farms allows us to conclude that these animals are the main contaminators of the nearby environment. In this study, we did not detect *Cryptosporidium* in the feces of human residents of these farms; this result may be due to various factors such as the small sample size of the population of susceptible humans (children and elderly), collection of human feces at time points that are different from those for collection of water, and the possibility that parasites identified in water were not capable of a clinical infection. Nevertheless, the identified species and strains and epidemiological characteristics observed in these environments pose a serious risk of contamination that may affect the population in question.

## Conclusions

In conclusion, the cattle of dairy farms in the region under study are infected with *Cryptosporidium* spp. and *Giardia* spp., with higher prevalence among neonates: calves up to 2 months of age. The presence of *Giardia* spp. in water samples may not be related to the excretion of cysts by the cattle because we did not identify assemblages of this parasite. Nevertheless, a zoonotic *C*. *parvum* strain was identified in water samples and feces from the animals of the same farm. Because fecal contamination was found in most water sources used for human and animal consumption on these farms, there is a need for a) assistance to the rural population regarding the management measures to reduce infection rates among the animals, b) preservation of water sources, and c) water treatment before consumption. The evidence seems to be sufficient to demonstrate the existing drawbacks in the water supply system of rural populations and the imminent risk to public and animal health.

## Supporting information

S1 Fig A flowchart of retrieval and analysis of fecal and water samples from 55 dairy farms in Paraná, Brazil, during 2012–2014. (PDF)

S1 Table Prevalence of *Giardia* spp and *Cryptosporidium* spp in feces of cattle from 55 dairy farms in Paraná, Brazil, from 2012 to 2014. ^**a**^Percentage calculated using the number of positive data from optical microscopy (OM). nPCR: nested PCR, 18S: 18S rRNA gene.
(PDF)

S2 Table Distribution of *Cryptosporidium* species by age groups according to genetic sequencing of fecal samples from 64 heads of cattle and PCR-RFLP of fecal samples from 15 heads of cattle from 28 dairy farms in Paraná, Brazil, from 2012 to 2014. ^**a**^nPCR: nested PCR, ^**b**^PCR-RFLP: restriction fragment length polymorphism, ^**c**^ ND: species not determined (ND) due to illegible sequence (sequencing data) or absence of DNA (PCR-RFLP), ^**d**^*C*. *parvum*, ^**e**^*C*. *bovis*.
(PDF)

S3 Table Variables with a statistically significant association with the presence of cysts of *Giardia* spp. and/or oocysts of *Cryptosporidium* spp. in fecal samples from 937 heads of dairy cattle in Paraná, Brazil, from 2012 to 2014. ^**a**^Fisher's exact test; ^**b**^OR: odds ratio; ^**c**^CI: confidence interval; ^**d**^*p*: probability.
(PDF)

S4 Table Prevalence of thermotolerant coliforms in 155 water samples obtained during the first visit and second visit to dairy farms in Paraná, Brazil, from 2012 to 2014. ^**a**^Thermotolerant coliforms according to the multiple-tube method; ^**b**^according to the chromogenic substrate method.
(PDF)

S5 Table Springhead, turbidity, bacteriological parameters of water samples positive for *Cryptosporidium* or *Giardia* species identified by genetic sequencing, and rainfall data 24 and 48 h prior to collection on four dairy farms in Paraná, Brazil, in 2014. ^**a**^Strongly concentrated by the flocculation method of calcium carbonate [29]; ^**b**^IFA: direct immunofluorescence assay; ^**c**^Oo/L: oocysts/liter of water; ^**d**^Cy/L: cysts/liter of water; ^**e**^ND: Not determined due to illegible sequence; ^**f**^nPCR: nested PCR; ^**g**^PCR-RFLP: restriction fragment length polymorphism; ^**h**^NTU: nephelometric turbidity unit; ^**i**^TTC: thermotolerant coliforms; ^**j**^MPN: most probable number.
(PDF)

S6 Table Variables with a statistically significant association with the presence of thermotolerant coliforms in 124 water samples from 55 dairy farms in Paraná, Brazil, from 2012 to 2014. ^**a**^OR: odds ratio; ^**b**^CI: confidence interval; ^**c**^*p*: probability.
(PDF)
