# Supplementary figures and images for: Cryptosporidium spp. and Giardia spp. in feces and water and the associated exposure factors on dairy farms
Source: PLoS One. 2017 Apr 12;12(4):e0175311. doi: 10.1371/journal.pone.0175311 (PMC5389815; doi:10.1371/journal.pone.0175311)

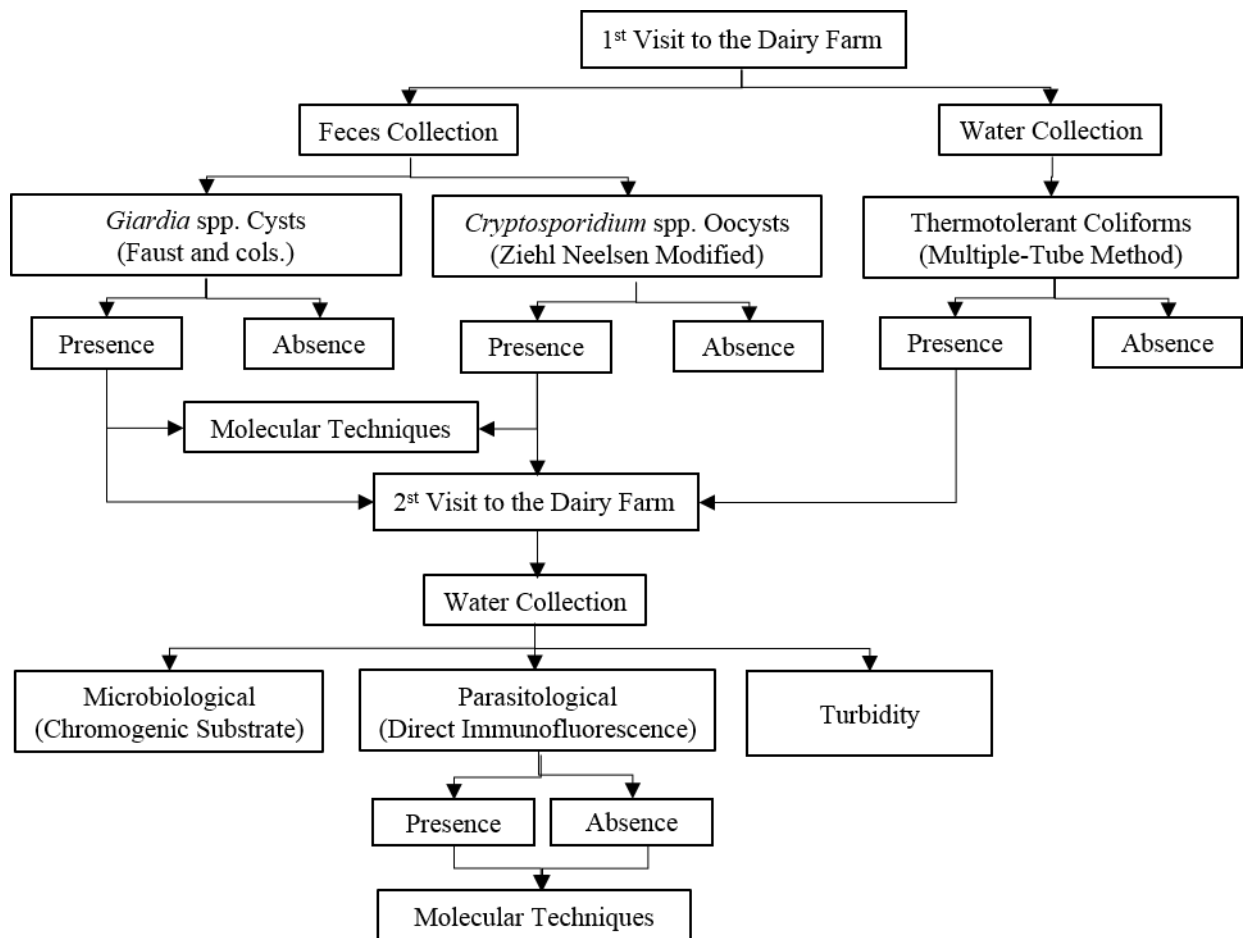

Supplement: S1 Fig — (PDF) [file pone.0175311.s001.pdf]
