# Supplementary material for: Cryptosporidium spp. and Giardia spp. in feces and water and the associated exposure factors on dairy farms
Source: PLoS One. 2017 Apr 12;12(4):e0175311. doi: 10.1371/journal.pone.0175311 (PMC5389815; doi:10.1371/journal.pone.0175311)
Supplement: S1 Table — aPercentage calculated using the number of positive data from optical microscopy (OM). nPCR: nested PCR, 18S: 18S rRNA gene. (PDF) [file pone.0175311.s002.pdf]

	Age Group (months)	Number of samples	*Giardia* spp.		*Cryptosporidium* spp.	
			**OM (%)**	***n*PCR 18S (%)[a]**	**OM (%)**	***n*PCR 18S (%)[a]**
0–6 months	0¬2	146	25 (17.1)	15 (60)	37 (25.3)	34 (91.9)
	2¬4	133	15 (11.3)	13 (86.7)	9 (6.8)	7 (77.8)
	4¬6	99	6 (6.1)	5 (83.3)	8 (8.1)	6 (75)
	Total (0 a 6)	378	46 (12.2)	33 (71.7)	54 (14.3)	47 (87)
> 6 months	6¬12	81	6 (7.4)	6 (100)	5 (6.2)	3 (60)
	12¬24	54	0	0	6 (11.1)	1 (16.7)
	>24	424	19 (4.5)	9 (47.4)	30 (7.1)	13 (43.3)
	Total (> 6 months)	559	25 (4.8)	15 (2.7)	41 (7.3)	17 (3.0)
	Total (All)	937	71 (7.6)	48 (67.6)	96 (10.2)	64 (66.7)
