# Supplementary material for: Cryptosporidium spp. and Giardia spp. in feces and water and the associated exposure factors on dairy farms
Source: PLoS One. 2017 Apr 12;12(4):e0175311. doi: 10.1371/journal.pone.0175311 (PMC5389815; doi:10.1371/journal.pone.0175311)
Supplement: S2 Table — anPCR: nested PCR, bPCR-RFLP: restriction fragment length polymorphism, c ND: species not determined (ND) due to illegible sequence (sequencing data) or absence of DNA (PCR-RFLP), dC. parvum, eC. bovis. (PDF) [file pone.0175311.s003.pdf]

Age Group (months)	DNA sequencing (%)						PCR-RFLP[b] *Cryptosporidium* spp. (%)				
	**Positive Samples (*n*PCR[a])**	***C. parvum***	***C. ryanae***	***C. bovis***	**ND [c]**	**Fragments of overlap**	***C. parvum/C. bovis***	***C. parvum/C. ryanae***	***C. parvum/C. andersoni***	**Single species**	**ND**
0¬2	33	16	0	0	4	13	2	2	0	5[d]	4
2¬4	8	4	1	0	1	2	0	1	1	0	0
4¬6	8	4	1	1	2	0	0	0	0	0	0
6¬12	3	1	0	0	0	2	0	0	1	1[e]	0
12¬24	0	0	0	0	0	0	0	0	0	0	0
>24	12	2	3	1	3	3	0	0	3	0	0
Total	64	27 (42.2)	5 (7.7)	2 (3.1)	10 (15.6)	20 (31.3)	2 (3.1)	3 (4.7)	5 (7.8)	6 (9.4)	4 (6,3)
