# Supplementary material for: Cryptosporidium spp. and Giardia spp. in feces and water and the associated exposure factors on dairy farms
Source: PLoS One. 2017 Apr 12;12(4):e0175311. doi: 10.1371/journal.pone.0175311 (PMC5389815; doi:10.1371/journal.pone.0175311)
Supplement: S3 Table — aFisher's exact test; bOR: odds ratio; cCI: confidence interval; dp: probability. (PDF) [file pone.0175311.s004.pdf]

Exposure variables	Cysts of *Giardia* spp.<br>Positive samples/Total (%)	OR[b]<br>(95% CI[c])	*p* [d]
**Production system**			
Business	10/63 (15.9)	2.51[a]	0.0318
Family	61/874 (7.0)	(1.08–5.31)	
**Lactating Cows**			
1 to 40	53/801 (6.6)	0.46	0.0117
>40	18/136 (13.2)	(0.26-0.82)	
**Feeding of Cows**			
Only trough	10/44 (22.7)	4.02[a]	0.002
Trough and pasture	61/893 (6.8)	(1.68–8.78)	
**Diarrhea frequency**			
High	41/346 (11.8)	2.51	0.0003
Low	30/591 (5.1)	(1.54–4.11)	
**Characteristics of feces (calves up to 2 months old)**			
Liquid/pasty	11/35 (31.4)	3.18<br>(1.28–7.87)	0.0203
Firm	14/111 (12.6)		
**Age Group (All)**			
0–6 months	46/377 (12.2)	2.97	0.0001
>6 months	25/560 (4.5)	(1.79–4.93)	
**Age Group (up to 6 months)**			
0–2 months	25/146 (17.1)	2.08	0.0296
2–6 months	21/232 (9.1)	(1.12–3.87)	
**Exposure variables**	**Oocysts of *Cryptosporidium* spp.**<br>**Positive samples/Total (%)**	**OR**<br>**(95% CI[c])**	***p* [d]**
**Breed**			
European	78/646 (12.1)	2.21	0.005
Zebu or Cross-bred Zebu	17/291 (5.8)	(1.29–3.81)	
**Characteristics of feces**			
Liquid/pasty	23/98 (23.5)	3.31	0.0001
Firm	72/839 (8.6)	(1.96–5.61)	
**Age Group (All)**			
0–6 months	54/95 (56.8)	2.12	0.0007
>6 months	41/559 (7.3)	(1.38–3.25)	
**Age Group (up to 6 months)**			
0–2 months	37/146 (25.3)	4.29	0.0001
2–6 months	17/232 (7.3)	(2.31–7.97)	
