# Supplementary material for: Cryptosporidium spp. and Giardia spp. in feces and water and the associated exposure factors on dairy farms
Source: PLoS One. 2017 Apr 12;12(4):e0175311. doi: 10.1371/journal.pone.0175311 (PMC5389815; doi:10.1371/journal.pone.0175311)
Supplement: S4 Table — aThermotolerant coliforms according to the multiple-tube method; baccording to the chromogenic substrate method. (PDF) [file pone.0175311.s005.pdf]

| Water sources | First Visit | | | Second Visit | | |
|---|---|---|---|---|---|---|
| | Number of sources | Number of samples | Positive Samples, TTC[a] (%) | Number of sources | Number of samples | Positive Samples, TTC[b] (%) |
| Spring | 31 | 62 | 35 (56.5) | 24 | 24 | 12 (50) |
| Artesian well | 20 | 40 | 3 (7.5) | 2 | 2 | 0 |
| Shallow well | 8 | 16 | 6 (37.5) | 2 | 2 | 0 |
| River | 3 | 6 | 6 (100) | 3 | 3 | 3 (100) |
| Total | 62 | 124 | 50 (40.3) | 31 | 31 | 15 (48.4) |
