# Supplementary material for: Cryptosporidium spp. and Giardia spp. in feces and water and the associated exposure factors on dairy farms
Source: PLoS One. 2017 Apr 12;12(4):e0175311. doi: 10.1371/journal.pone.0175311 (PMC5389815; doi:10.1371/journal.pone.0175311)
Supplement: S5 Table — aStrongly concentrated by the flocculation method of calcium carbonate [29]; bIFA: direct immunofluorescence assay; cOo/L: oocysts/liter of water; dCy/L: cysts/liter of water; eND: Not determined due to illegible sequence; fnPCR: nested PCR; gPCR-RFLP: restriction fragment length polymorphism; hNTU: nephelometric turbidity unit; iTTC: thermotolerant coliforms; jMPN: most probable number. (PDF) [file pone.0175311.s006.pdf]

Property	Source of water	*Giardia* spp.				*Cryptosporidium* spp.				Turbidity (NTU)[h]	TTC[i] (MPN[j]/ 100mL)	Rainfall prior to collection (mm)	
		IFA[b] (Cy/*L*)[d]	*n* PCR[f]	Sequencing	Positive Cattle	IFA[b] (Oo/*L*[c])	*n* PCR[f]	Sequencing/ [g]PCR-RFLP	Positive Cattle			24 h	48 h
26	Spring	0	(-)	-	Yes	1.3	(+)	*C. parvum*	Sim	36.4	1046	83	76
27 [a]	River	12	(+)	ND[e]	No	0	(+)	*C. parvum/ C. andersoni*	Não	98.5	2419.5	44	0
34	Spring	0.34	(+)	*G. duodenalis*	Yes	0.34	(+)	*C. parvum/ C. andersoni*	Sim	25.6	24.5	43	0
44	Spring	2.1	(+)	*G. duodenalis*	Yes	0	(-)	-	Sim	64.47	2419.6	182.4	0
