# Supplementary material for: Cryptosporidium spp. and Giardia spp. in feces and water and the associated exposure factors on dairy farms
Source: PLoS One. 2017 Apr 12;12(4):e0175311. doi: 10.1371/journal.pone.0175311 (PMC5389815; doi:10.1371/journal.pone.0175311)
Supplement: S6 Table — aOR: odds ratio; bCI: confidence interval; cp: probability. (PDF) [file pone.0175311.s007.pdf]

Variables	Thermotolerant Coliforms<br>Positive samples/Total (%)	OR[a] (95% CI[b])	*p*[c]
**Source of Water**			
River	6/6 (100)	-	0.0001
Spring	35/62 (56.45)	15.99 (4.45–57.45)	0.0001
Shallow well	9/60 (15)	7.4 (1.57–34.93)	0.01294
Artesian well	3/40 (7.5)	1	
**Collection site**			
Source	22/37 (59.46)	3.09 (1.4–6.85)	0.0084
Water tank/Tap	28/87 (32.2)		
**Slapping source**			
Yes	36/103 (34.95)	0.27 (0.08–0.8)	0.0074
No	14/21 (66.7)		
**Rainfall prior to collection (up to 48 h)**			
Yes	25/46 (54.3)	2.52 (1.2–5.34)	0.0241
No	25/78 (32.1)		
**Spring with side protection**			
Yes	18/40 (45)	0.24 (0.074–0.78)	0.02892
No	17/22 (77.3)		
**Spring with riparian forest**			
Yes	19/42 (45.2)	0.21 (0.06–0.72)	0.02107
No	16/20 (80)		
